# Capecitabine/Mitomycin versus 5-Fluorouracil/Mitomycin in Combination with Simultaneous Integrated Boost Intensity-Modulated Radiation Therapy for Anal Cancer

**DOI:** 10.3390/curroncol30090621

**Published:** 2023-09-18

**Authors:** Laurent Mineur, Léa Vazquez, Mohamed Belkacemi, Clémence Toullec, Newfel Bentaleb, Rania Boustany, Frederi Plat

**Affiliations:** 1 Oncodigestive and Clinical Research Department, Sainte Catherine Institut du Cancer Avignon-Provence, 84918 Avignon, France; 2 Statistics Department, PRECIS, Nouvelles Technologies, Languedoc Mutualité, 34000 Montpellier, France

**Keywords:** anal cancer, capecitabin, 5-FU, radiation therapy, toxicity

## Abstract

Since EXTRA, a non-randomized phase II trial with 31 patients, explored the use of capecitabine, mitomycin and radiation therapy (RT) in the treatment of localized squamous cell carcinoma of the anal canal (SCCAC), this treatment has been considered as an acceptable alternative to infusional 5-FU. However, the differences in efficacy between capecitabine and 5-FU in chemoradiation therapy (CRT) with simultaneous integrated boost (SIB) radiation therapy (SIB-IMRT) for local SCCAC are not well documented. Patients included in this prospective monocentric cohort study were treated with SIB-RapidArc (a unique RT method treatment for all patients: identical technique, volume and constraints for at-risk organs), mitomycin C and 5-FU each day of RT for 7 weeks (group 1) or capecitabine each day of RT (group 2). Patients treated between July 2009 and August 2017 (group 1) and between November 2012 and April 2018 (group 2) for local SCCAC T2-4 classified as N, M0 or T, N1-3, M0 were included. Primary endpoints were progression-free survival (PFS) and acute toxicities. Results: One hundred forty-seven patients were included, 91 in group 1 and 56 in group 2. The two groups were statistically comparable in terms of sex, Eastern Cooperative Oncology Group Performance Status (ECOG PS) and TNM. With a median duration of follow-up of 53.5 months, the PFS rate at 3 years was 80% for group 1 and 75% for group 2 (*p* = 0.32). The 3-year colostomy-free survival rate was 92% for group 1 and 85% for group 2 (*p* = 0.11). The rate of patients with at least one grade 3 or higher acute toxicity was 35.5% in group 1 and 21.4% in group 2 (*p* = 0.10), with a trend of fewer acute toxicities with capecitabine. Conclusion: Capecitabine/mitomycin in combination with SIB RapidArc radiation therapy for anal cancer seems as effective as 5-FU-based chemotherapy and is well tolerated with minimal toxicity.

## 1. Introduction

Squamous cell carcinoma of the anal canal (SCCAC) is a relatively rare cancer, with approximately 14,500 cases in women and 12,500 in men occurring in 2008 worldwide, and represents 2% of all cancers in Europe [1,2]. Several studies have reported an increase in the incidence rate in some very-high-income countries, including France [3].

Multimodal therapy with chemotherapy and radiation is the cornerstone of anal cancer treatment, with surgery generally reserved for those who have disease progression despite chemoradiation. Several trials have examined the optimal regimen for patients with locally advanced disease [4,5,6,7] and demonstrated that radiotherapy with concomitant 5-fluorouracil (5-FU) and mitomycin C (MMC) resulted in superior outcomes in terms of disease-free survival (DFS) and sphincter preservation. Based on these studies’ findings, chemoradiotherapy remains the preferred treatment for most patients. Nowadays, the standard of care is infusional 5-FU 1000 mg/m2/day for 4 or 5 days in weeks 1 and 5 of radiotherapy as a radiosensitizer and MMC 12 mg/m2 as a bolus on day 1 combined with full-dose radiation therapy [8].

Radiotherapy is usually applied using a two- or three-field technique to a total dose of 45–50.4 Gy in 4–5 weeks, sometimes followed by a boost up to 59.4 Gy [5,7,9]. However, with this conventional radiation therapy technique, concurrent chemoradiation is associated with relevant acute toxicities leading to treatment breaks, thereby increasing the risk for local tumor recurrence [10,11]. The use of intensity-modulated radiation therapy (IMRT) compared to conformal therapy was associated with decreased toxicity, a consequent reduction in treatment interruptions and similar outcomes in terms of local control and survival [12,13,14,15]. RapidArc (RA) is a specific form of volumetric modulated arc therapy (VMAT), a method combining rotational therapy techniques with intensity modulation, delivering a precisely sculpted 3D dose distribution with a 360° rotation of the accelerator gantry due to a treatment planning algorithm that simultaneously varies gantry rotation speed, movement of the multileaf collimator and delivery dose rate [16,17].

The use of capecitabin to replace 5-FU is another potential improvement in the treatment of SCCAC. In gastrointestinal cancers, including colorectal cancer, given their equivalent outcome, the replacement of 5-FU with capecitabine is increasingly important. Indeed, to act as a radiosensitizer, 5-FU should be administered in the form of prolonged intravenous infusion during radiotherapy, which is a constraint for patients [18]. Therefore, the twice-daily administration of capecitabine, converted into 5-FU in the gastrointestinal tract by thymidine phosphorylase, itself upregulated by radiation, allows continuous exposure to the active drug [19]. The oral administration of capecitabine mimics the pharmacokinetics of infusional 5-FU and offers greater convenience to patients, avoiding central catheter insertion and/or hospitalization. Randomized studies comparing capecitabine to infusional 5-FU have been undertaken in colorectal and gastric cancers and have shown the non-inferiority of capecitabine [20,21].

The role of capecitabine in SCCAC was investigated in phase II trials and several retrospective studies [22,23,24,25,26,27,28], but the differences in efficacy between capecitabine and 5-FU in combination with simultaneous integrated boost (SIB) RapidArc radiation therapy for local SCCAC are not well documented and need confirmation. 

## 2. Materials and Methods

### 2.1. Study Population

All patients ≥18 years of age with histologically confirmed squamous locally advanced anal cancer, classified as T1-4, N2-3, M0 or T2-4, N0-1, M0, treated at our institute between July 2009 and October 2018 with concurrent chemoradiotherapy were included. All patients who received 5-fluorouracil (5-FU) were included in group 1 (between July 2009 and August 2017), and all patients who received capecitabine were included in group 2 (between November 2012 and October 2018). 

Disease staging was performed via rectal exam, magnetic resonance imaging (MRI), and/or echo-endoscopy (EUS) and PET scanning, according to the American Joint Committee on Cancer (AJCC) manual and the International Union Against Cancer 5IUAC) system. The study was approved by the local ethics committee and was conducted in accordance with Good Clinical Practice guidelines.

### 2.2. Study Design

This study was designed as a monocentric prospective cohort study. The objectives were to determine the efficacy and safety of SIB RapidArc with concomitant capecitabine and mitomycin C (MMC) compared to SIB RapidArc with concomitant 5-FU and mitomycin C in patients with locally advanced anal cancer. 

Toxicity was graded using the National Cancer Institute Common Terminology Criteria for Adverse Events (NCI-CTCAE) version 4.0. During treatment, acute toxicity was recorded during weekly clinic visits with the radiation oncologist. Acute toxicities were scored at the worst grade occurring from the start of treatment until 30 days after the last fraction of radiotherapy. 

Tumor measurement at baseline included physical examination, rectal examination, computed tomography (CT) of the chest and abdomen, magnetic resonance imaging (MRI) of the pelvis or echo-anoscopy and whole-body 18F-FDG positron emission tomography (PET)-CT. 

Tumor response was evaluated by rectal examination during the treatment and by PET-CT and rectal endoscopy with biopsies 3 months after the completion of treatment. Complete response was defined as the absence of any sign of residual disease in imaging and histology if necessary.

Locoregional recurrence was defined as the recurrence or persistence of disease locally or elsewhere in the pelvic or inguinal nodes. Distant metastasis was defined as the development of disease outside the pelvic or inguinal lymph nodes.

Clinical evaluation was performed weekly during the treatment, including assessment of adverse events, physical examination, Eastern Cooperative Oncology Group Performance Status (ECOG PS) and vital signs. After that, the clinical evaluation was performed every 3 months after the completion of treatment for 2 years, every 6 months over the next 3 years, and then every year. 

### 2.3. Study Treatment

#### 2.3.1. Radiotherapy

Patients were treated with SIB RapidArc radiation therapy. A total radiation dose of 59.4 Gy was delivered in 33 fractions of 1.8 Gy to the primary tumor and macroscopically involved lymph nodes and/or PET-CT positive nodes. The selectively treated lymph nodes received a total dose of 49.5 Gy in 33 fractions of 1.5 Gy in the same overall treatment time. A boost dose was given sequentially to the primary tumor and macroscopically involved lymph nodes without a treatment gap for 14 patients (8.9%), whereas all other patients received simultaneous integrated boost (*n* = 143, 91.1%). 

Regarding radiation therapy data, we recorded the total dose received, the total number of therapy days, radiation treatment interruptions >3 days, and if necessary, the duration and the reason for these treatment interruptions. 

#### 2.3.2. Chemotherapy

Chemotherapy consisted of mitomycin C 10 mg/m2D1D28 + 5-FU, given as a continuous infusion of 250 mg/m2 daily of radiation therapy for 7 weeks, for group 1 patients. Group 2 patients received mitomycin C 10 mg/m2D1D28 + capecitabine 825 mg/m2 BID each day of radiation therapy (RT). Group 1 patients were treated between July 2009 and August 2017, and group 2 patients were treated between November 2012 and April 2018. In 2013, a phase I study was published using capecitabine instead of 5-FU and reported the safety and effectiveness of capecitabine with SIB-IMRT for locally advanced canal cancer [23]. After this publication, patients began to receive capecitabine, except patients with renal dysfunction. As this phase I study was initiated in February 2008 and the results were already known in August 2012 (first submission), some older patients began to receive capecitabine in November 2012. 

### 2.4. Statistical Analysis

Descriptive statistics are presented as medians and ranges for quantitative variables. Discrete variables are reported as counts and percentages. The Wilcoxon rank sum test was applied to compare the distribution of continuous variables, and the chi-squared test (or Fisher’s exact test when appropriate) was used to test the association of categorical variables.

The primary endpoints were progression-free survival (PFS) and acute toxicities. Colostomy-free survival (CFS) and overall survival (OS) were secondary endpoints. 

PFS was defined as the time from the date of diagnosis to the date of disease progression, date of death (due to any cause) or date of last follow-up. OS was defined as the time from the date of diagnosis to the date of death (due to any cause) or last follow-up. CFS was defined as the time from the date of diagnosis to the date of colostomy or last follow-up. Estimates of PFS, OS and CFS were obtained using the Kaplan–Meier method, and the log-rank test was used to compare differences between survival curves. The median follow-up time was computed using the inverse Kaplan–Meier method.

SAS software version 9.4 was used for all statistical analyses.

## 3. Results

### 3.1. Patient and Treatment Characteristics

A total of 157 patients were included in the study between July 2009 and October 2018, 95 in group 1 and 62 in group 2. The median age at diagnosis was 64 (range: 38–93), and most of the patients were female (82.8%). Four patients (two in group 1 and two in group 2) had HIV (human immunodeficiency virus)-positive status. All tumors (100%) were squamous cell carcinoma of the anal canal (SCCAC). The patient characteristics are summarized in Table 1. There were no significant differences between the two groups except for median age (61 vs. 67, *p* < 0.005). Patients who received capecitabine were statistically older than patients who received 5-FU. The median follow-up was also longer for group 1 (67.4 vs. 41.5 months).

Radiotherapy was completed without interruptions in 82.11% of the group 1 patients vs. 87.1% of the group 2 patients (*p* = 0.51). The radiation dose was delivered within a median duration of 52 days (range: 43–79). Twenty-five patients underwent a break of more than three days during their treatment, with a median break duration of six days (range: 4–25). The causes of stoppage were digestive (44%, *n* = 11), hematological (24%, *n* = 6), planning (20%, *n* = 5), radiation-induced adverse effects (16%, *n* = 4) and other causes unrelated to radiation therapy (8%, *n* = 2).

The complete chemotherapy dose was delivered to 56.8% of group 1 patients vs. 46.8% of group 2 patients (*p* = 0.25). There was no significant difference in compliance with the treatment plan.

### 3.2. Toxicities

Toxicities were moderate in both groups (Table 2). All patients in both groups were assessable for toxicities. Toxicities were graded according to the National Cancer Institute Common Terminology Criteria for Adverse Events (NCI-CTCAE) version 4.0. The most common grade 3–4 toxicities encountered were diarrhea (group 1 7.4% vs. group 2 12.9%, *p* = 0.27), followed by dermatitis (7.8% vs. 4.0%, *p* = 0.49) and anitis (6.3% vs. 6.8%, *p* = 1). Grade 3–4 hematological toxicity was detected in eight patients (12.9%) in group 1 and two patients (10.5%) in group 2. Grade 3–4 digestive toxicity was detected in 16 patients (17.2%) in group 1 and 11 patients (18.6%) in group 2. Seventeen patients (18.5%) in group 1 and three patients (5.2%) in group 2 suffered due to other toxicities like renal failure, asthenia, vaginitis, cystitis, epithelitis or alopecia. Group 1 patients experienced more other toxicities than group 2 patients (*p* = 0.03). Chemotherapy dose reduction was performed for 38 patients (40%) in group 1 and 28 patients (45.1%) in group 2 (*p* = 0.62).

### 3.3. Outcomes and Survival

The median follow-up from the time of initial diagnosis was 53.5 months (range: 4.6–120.9).

Thirty-four (21.7%) of the patients had a recurrence. Thirteen patients (8.3%) had only local recurrence, 11 (7%) had local and distant metastases and 10 patients (6.4%) had only distant metastases.

**Figure 1:**
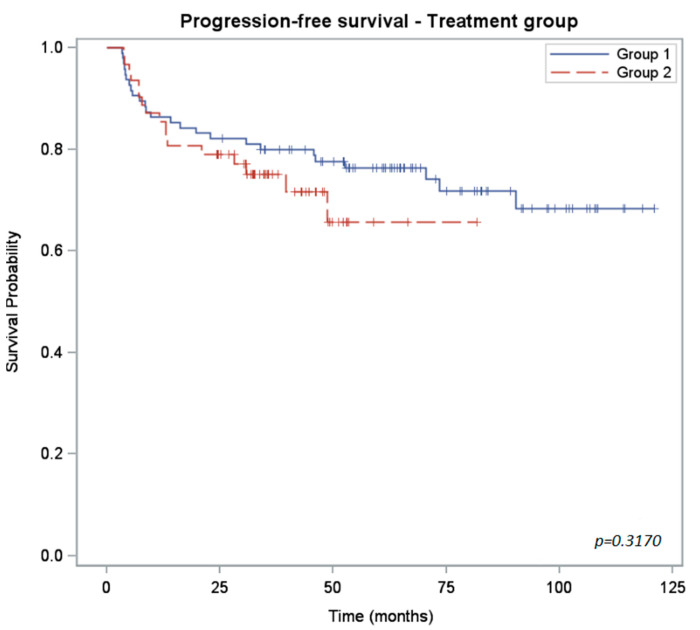
Kaplan–Meier curves of progression-free survival for 5-FU-treated patients (group 1) versus capecitabine-treated (group 2) patients.

**Figure 2:**
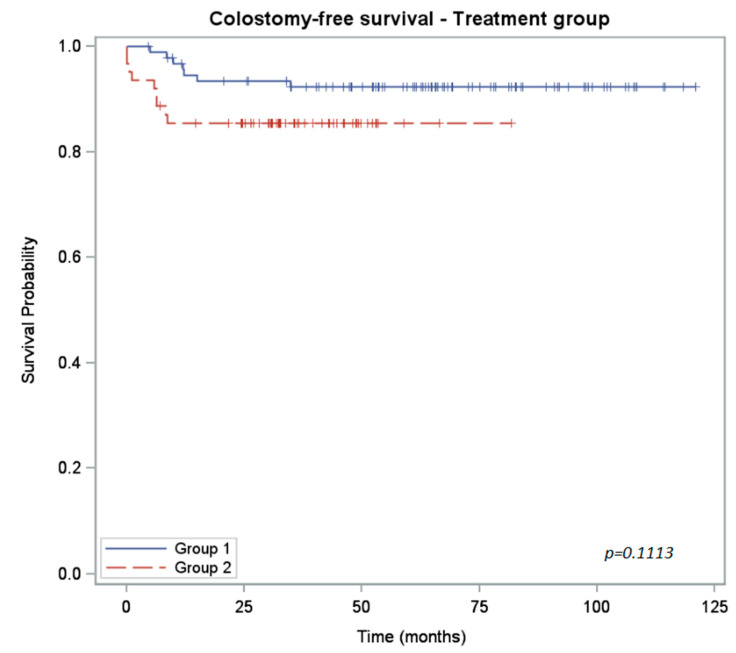
Kaplan–Meier curves of colostomy-free survival for 5-FU-treated patients (group 1) versus capecitabine-treated (group 2) patients.

**Figure 3:**
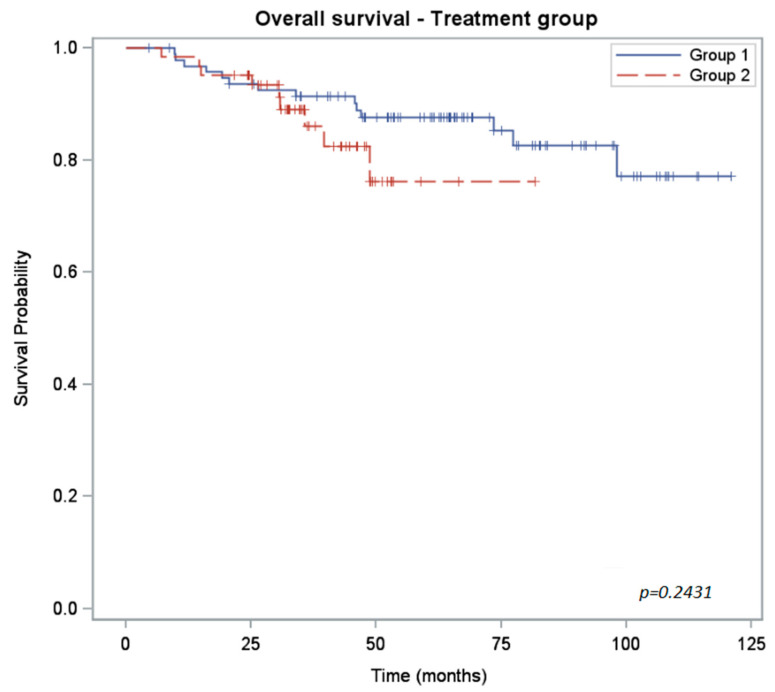
Kaplan–Meier curves of overall survival for 5-FU-treated patients (group 1) versus capecitabine-treated (group 2) patients.

The Kaplan–Meier plots of progression-free survival (PFS), overall survival (OS) and colostomy-free survival (CFS) rates are shown in Figure 1, Figure 2 and Figure 3. The PFS rate at 3 years was 80% for group 1 and 75% for group 2 (*p* = 0.32). The 3-year OS rate was 91% for group 1 and 86% for group 2 (*p* = 0.24). Because of the shorter follow-up for the group 2 cohort, we anticipate that the number of locoregional and distant recurrences in group 2 may increase over time.

With 8.6% and 7.1% of patients requiring salvage abdominoperineal resection (APR) in group 1 and group 2, respectively, there is no significant difference for this point between the two groups (*p* = 1.00).

The 3-year colostomy-free survival rate was 92% for group 1 and 85% for group 2 (*p* = 0.11).

The differences between the two groups for each survival measure are shown in Table 3.

### 3.4. Comparison of Efficacy and Toxicities with Previous Studies

Despite the comparable results in both groups with most studies, the PFS in both groups in our study seemed to be slightly lower than the PFS in the studies of Deenen et al. and Pumpalova et al., while both studies also used IMRT [23,28]. Concerning the incidence of grade 3–4 toxicities (dermatitis, gastrointestinal and hematological), the results were like those of other studies, despite our group showing a slight decrease in grade 3–4 dermatitis compared to others.

## 4. Discussion

In this prospective comparative study, we have found comparable outcomes between intensity-modulated radiation therapy (IMRT) and mitomycin C plus oral capecitabine or infusional 5-FU in locally advanced squamous cell carcinoma of the anal canal (SCCAC). The 3-year progression-free survival (PFS), 3-year colostomy-free survival (CFS) and disease-specific survival (DSS) rates were comparable between the two groups. Both treatment regimens were well tolerated, with few grade 3–4 acute toxicities.

Capecitabine was developed as a potential substitute for 5-FU because of its ease of administration. Indeed, although the risk of venous catheter-related toxicity associated with infusional 5-FU is low, patient comfort and treatment tolerability are important factors to consider in recommending oral capecitabine as a substitute for infusional 5-FU [29,30]. Additionally, daily capecitabine associated with radiation therapy imitates prolonged low-dose infusional 5-FU, seems to be associated with lower hematological toxicities and may be better tolerated overall than 5-day infusional 5-FU [31]. In several studies on rectal cancer, capecitabine has shown equivalent efficacy to infusional 5-FU but with a milder toxicity profile and better patient tolerability.

A similar substitution is recommended in the treatment of anal cancer, even if, currently, there are no available randomized phase III data to confirm equivalent efficacy between oral capecitabine plus mitomycin (MMC) and radiation therapy and infusional 5-FU plus MMC and radiation therapy in anal cancer. Several single-institutional reviews have shown comparable outcomes and toxicity profiles between capecitabine and infusional 5-FU. The main toxicity of chemoradiotherapy treatment for locally advanced anal cancer is radiation dermatitis. The radiation dermatitis rate with capecitabine in these studies ranged between 23% and 63%. Grade 3–4 diarrhea and hematological toxicity rates ranged from 0 to 17% and 0 to 60%, respectively [21,24,28].

In the first phase II trial in which capecitabine was combined with conventional radiotherapy, and applied without a treatment gap, a comparable rate of grade 3–4 gastrointestinal and hematological toxicities was found, but the rate of grade 3–4 dermatological toxicities (38%) was more important than that in our study (9%). The 6-month PFS (94%) was also comparable to that in our study (93%). The CFS was not considered in this study [22]. In the retrospective study by Meulendjiks et al., both capecitabine and 5-FU arms in combination with RT had an acceptable toxicity profile, although dermatitis toxicity was more important than that in our study (31% and 9%, respectively) [27]. The PFS was not considered in this study, but comparable outcomes in terms of OS and locoregional control were found between the two patient cohorts. The same year, another retrospective study described the toxicity, dose intensity and outcomes of a sequential cohort of patients treated with chemoradiotherapy with capecitabine (mainly 3D-chemoradiation therapy without boost). In the 66 patients retrospectively reviewed and followed for 20 months, the treatment was well tolerated and allowed a high dose intensity of radiation and chemotherapy, although dermatitis toxicity was particularly important (63%) [25]. In the second phase II trial in which capecitabine was used in substitution of 5-FU in the chemoradiotherapy regimen for patients with locally advanced SCCAC, capecitabine offered similar efficacy to infusional 5-FU and provided manageable toxicity. However, it should be noted that hematological toxicities were significantly higher in patients receiving IMRT (60%) than in patients receiving 3D-CRT (20%) [26]. Although all patients included in our study received IMRT as radiation therapy, the hematological toxicity did not exceed 6% in our population.

In their retrospective study, Peixoto et al. analyzed the impact of regimen on disease-free survival (DFS) and anal-cancer-specific survival (ACSS) among patients with stage I-III anal cancer treated either with capecitabine/MMC/RT or 5FU/MMC/RT [24]. They showed similar DFS and ACSS between their two cohorts, with progression-free survival like that in our study (2-year PFS = 80%). Unfortunately, the safety of the two treatments was not compared in this study [24]. On the other hand, a few months later, Goodman et al. published a retrospective study on a cohort of patients treated with a continuous course of chemoradiation using IMRT for all patients to a standard dose of 50 to 56 Gy with concurrent MMC and either 5-FU or capecitabine. In their study population, pelvic radiation therapy with MMC plus capecitabine was well tolerated and appeared to have less grade ≥3 acute hematologic toxicity and fewer treatment interruptions than a historical control group receiving 5-FU [31].

The most recent retrospective study published in which capecitabine and 5-FU were compared in association with MMC and radiation therapy in all non-metastatic anal cancer patients found comparable outcomes (OS, ACSS and incidence of recurrence) between the two patient cohorts. Severe radiation dermatitis and hematological toxicities were common in both groups, but the treatment was well tolerated [28].

Our study, also single-institutional, used a prospective design. The monocentric conception could limit the generalizability of the findings to a broader population. However, our findings and conclusion were like the results reported in these other studies which support the use of capecitabine in the treatment of anal cancer. Another weak area of the study is the difference in follow-up duration, which could impact the accuracy of comparing outcomes. Therefore, the number of locoregional and distant recurrences in group 2 may increase over time. So, the non-inferiority in outcomes could be ruled out with a longer follow-up and/or a larger sample size. Indeed, the patterns of progression in our patients receiving capecitabine appear to tend towards an increased rate of progression over time compared to our patients receiving 5-FU. This trend, although not statistically significant with our follow-up, could become so upon an increase in the follow-up duration and/or the sample size. It could be interesting to continue monitoring group 2 patients.

Another study limitation was the uncontrolled imbalance between the two patient cohorts in this study. Patients in the cohort receiving capecitabine (group 2) were significantly older than patients receiving 5-FU (group 1). This demographic difference could introduce bias and confounding effects that might affect the study’s outcomes. 

Ultimately, these data support the consideration of capecitabine as an alternative radiosensitizer option for anal cancer. It will be difficult to undertake a randomized controlled trial directly comparing capecitabine and 5-FU in chemoradiation therapy of locally advanced SCCAC due to the low incidence of anal cancer and difficulty recruiting patients for a randomized trial.

## 5. Conclusions

Chemoradiotherapy with capecitabine and MMC appears to be equal in efficacy to infusional 5-FU and MMC in locally advanced SCCAC. Their acute toxicity profiles are comparable.

## Figures and Tables

**Table 1:** Patients and treatment characteristics.

	5-FU-Based CT Treatment *N* = 95		Capecitabine-Based CT Treatment *N* = 62		*p*-Value
Characteristics	*n*	%	*n*	%	
Age (years), median (range)	61 (38–90)		67 (39–93)		0.0048
Sex Male Female	17 78	17.89 82.11	10 52	16.13 83.87	0.8316
ECOG performance status 0 1–2	91 4	95.79 4.21	57 5	91.94 8.06	0.3192
History of pelvis/obstetric surgery Yes No/unknown	49 46	51.58 48.42	25 37	40.32 59.68	0.1925
T-classification 1–2 3 4	60 22 13	63.16 23.16 13.68	36 19 7	58.06 30.65 11.29	0.5583
N-classification 0 1 2–3	41 21 33	43.16 22.10 34.74	31 12 19	50.00 19.35 30.65	0.7389
HPV status Negative Positive Missing	3 25 67	3.16 26.31 70.53	3 36 23	4.84 58.06 37.10	**<0.001**
HIV status Negative Positive Missing	21 2 72	22.10 2.11 75.79	29 2 31	46.77 3.23 50.00	**0.0020**
SCC tumor marker Normal Elevated Unknown	35 23 37	36.84 24.21 38.95	25 14 23	40.32 22.58 37.10	0.9342
Radiation technique: boost-integrated Yes No	86 9	90.53 9.47	57 5	91.94 8.06	1.0000
Median RT treatment days (range)	52 (46–78)		52 (43–79)		0.7949
RT interruptions > 3 days Yes No	17 78	17.89 82.11	8 54	12.90 87.10	0.5053
Median duration of RT interruption (range), days	6 (4–25)		9.5 (5–20)		0.0203
Chemotherapy dose reduction Yes No	38 57	40.00 60.00	28 34	45.16 54.84	0.6200
Capecitabine or 5FU dose received <50% 50–75% 75–100% 100%	1 12 25 57	1.05 12.63 26.32 60.00	6 10 12 34	9.68 16.13 19.35 54.84	0.0641

**Table 2:** Most common acute toxicities.

Toxicity	5-FU-Based CT Regimen					Capecitabine-Based CT Regimen				
	Grade					Grade				
	Number of Patients * N = 95	1	2	3	4	Number of Patients * N = 62	1	2	3	4
Hematological toxicities										
Anemia	33	23	8	2	0	11	8	3	0	0
Thrombopenia	50	40	6	4	0	12	7	3	1	1
Neutropenia	24	17	4	2	1	6	4	1	1	0
Digestive toxicities										
Diarrhea	73	31	35	7	0	47	22	17	8	0
Nausea	27	21	5	1	0	11	8	3	0	0
Anorexia	28	22	5	1	0	13	7	5	1	0
Vomiting	6	3	1	2	0	2	1	0	1	0
Rectitis	43	25	17	1	0	25	15	10	0	0
Anitis	80	28	47	5	0	44	22	19	3	0
Other toxicities										
Renal failure	6	5	1	0	0	0	0	0	0	0
Asthenia	51	23	22	6	0	26	15	11	0	0
Vaginitis	26	17	8	1	0	12	10	1	1	0
Cystitis	32	24	8	0	0	7	7	0	0	0
Dermatitis	77	37	34	6	0	50	31	17	2	0
Alopecia	20	2	14	4	0	0	0	0	0	0

* Some patients experienced more than one toxicity.

**Table 3:** Survival.

	5-FU-Based CT Treatment *N* = 95	Capecitabine-Based CT Treatment *N* = 62	*p*-Value
Follow-up median (months) CI_95%_	67.44 (63.51; 78.03)	41.51 (34.59; 46.26)	/
PFS rate (%) CI_95%_	2 years: 82 (73; 88) 3 years: 80 (70; 87)	2 years: 79 (67; 87) 3 years: 75 (62; 84)	0.3170
OS rate (%) CI_95%_	2 years: 94 (86; 97) 3 years: 91 (83; 96)	2 years: 95 (86; 98) 3 years: 86 (72; 93)	0.2431
CFS rate (%) CI_95%_	2 years: 93 (86; 97) 3 years: 92 (85; 96)	2 years: 85 (74; 92) 3 years: 85 (74; 92)	0.1113

CFS = colostomy-free survival.

## Data Availability

The datasets used and/or analyzed during the current study are available from the corresponding author on reasonable request.
